# On Dr. Brown-Séquard and His Recent Lectures

**Published:** 1878-07

**Authors:** J. S. Jewell

**Affiliations:** Professor of Nervous and Mental Diseases in the Chicago Medical College


### Chicago Medical Journal

AND

### EXAMINER.

Vol. XXXVII.—JULY, 1878.—No. 1.

### Original Lectures.

### ON DR. BROWN-SÉQUARD AND HIS RECENT LECTURES.

A Lecture delivered before the Chicago Medical Society, May 6th, 1878.

By J. S. Jewell, M. D.,

(*Professor of Nervous and Mental Diseases in the Chicago Medical College.*)

(CONCLUDED.)

Mr. President and Members of the Society:

But one of the most striking classes of pathological proof of the decussation of the motor tract, is found in the now rather well known "secondary degenerations," first described by Tuerck, of Vienna, (*Ueber die second. Erkrankungen einzelner Rueckens-marks-strange u. ihre Fortsetzungen zu gehirn*, *Wien. akad. Sitz. Ber. Meth. Cl. Bd.* 6 *Jahrg.* 1851. 1 *Haelfte*, *Seite* 288 *fg.; Bd.* 11. *Jahrg.* 1853, 2 *Haelfte*, *Seite* 93, *fg.*,) and later by Charcot, Erb, Berger, and many others.

In this case it is rendered pretty certain that disease starting within the limits of what has been called the "motor zone" of the brain, will descend the motor tract until it reaches the level of the pons and medulla, and then still following, apparently, the continuous motor tract, it *passes over to the opposite side of the cord*, and in that side descends. These cases can be easily explained on the view that the motor tracts decussate, as has been claimed, but not on the hypothesis of Dr. Brown-Séquard. These cases of "secondary degeneration" in reality powerfully support the hypothesis of a decussation of the motor tracts. I cannot go farther this evening into this very extensive subject; but I cannot pass on, without recalling what seems to me to be a fact, that all the evidence worthy of the name, whether anatomical, physiological or pathological, supports this view. But while this is so, I must confess that I have been always favorably inclined to the view of Dr. Brown-Séquard as respects the non-decussation of the motor tracts. I have often asked myself the question, *why* there should be such a condition. Why should not the motor fibers from one side of the brain go to the same side of the body? What benefit can arise from the crossed arrangement which could not be secured in a simpler manner? I have never yet been able to answer these questions for myself. But so long as the evidence shows so plainly that there *is* a decussation of motor conductors, so long will I admit it as a fact.

2. *Localization of function in the cerebral cortex.*

It was not until the time of Gall that any considerable progress was made in a knowledge of the inner structure of the brain. Whatever may be said of his vagaries in craniscopy and phrenology, he gave a great impulse to cerebral anatomy and physiology. But his crude and ill-digested notions met with a vigorous repulse from many quarters, especially from Flourens and his disciples, who rejected the doctrine of a localization of function in the brain, (more particularly the cortex,) in favor of an opposite form of doctrine, or that of a diffusion of special functions throughout the cortex. This latter doctrine affirms in substance that the nervous cells in the cortex—for the sake of an example—which cause remotely the motion of a muscle, are not located in any particular part of the brain, but are scattered throughout the cortex, widely distant from one another. The cells of the cortex, which are remotely concerned in moving the arm, are not found in one cluster in one convolution, but they are scattered, some in this, and some in that one. Hence destruction of any one part of the cortex, while it may embarrass, cannot destroy or paralyze the movements of the arm. This is the view of Dr. Brown-Séquard. He utterly opposes the doctrine of a localization of function in the cortex of the brain. In order to show this, I will quote a passage from his lectures in this city.

He says (*Journal and Examiner*, p. 294, March, 1878,) "Contrary to what is now admitted, as regards instances of certain so-called psycho-motor centers, I will try to show that an agglomeration or cluster of cells located in * * * the brain, (the cells employed in moving the arm, for instance, and serving therefore for a center,) I will try to show, that such an agglomeration of cells does not exist anywhere, and that the cells which are employed in moving the arm for instance, are scattered all over the brain, so that destruction may take place in any part of the brain, without any paralysis of the arm; all these cells however, serving to move the one limb, or any other part, etc."

Many more such passages might be cited, and they would all contribute to make it clear that the view referred to of Dr. Brown-Séquard has been correctly described above. The proofs of a localization of function in the cortex of the brain, are of various kinds. I cannot go into these proofs, such as they are in detail, even if it were necessary, for it must be supposed most of those present are in a measure acquainted with them. But I cannot pass on without referring briefly to them.

Then, first of all, the doctrine is not only in harmony with, but rendered imperative, by the general principles of physiology. I do not know that this view can be better stated than it has been by Mr. Spencer in his work on psychology (vol. I. 573–4). I quote as follows:

"Whoever," says he, "calmly considers the question, cannot long resist the conviction that different parts of the cerebrum must, *in some way or other*, subserve different laws of mental action. Localization of function is the *law of all organization whatever*, and it would be marvelous were there here an exception. If it be admitted that the cerebral hemispheres are the seats of the higher psychical activities, if it be admitted that among these higher psychical activities there are distinctions of kind, which, though not definite, are yet practically recognizable, it cannot be denied, without going in direct opposition to established physiological principles, that these more or less distinct kinds of psychical activity must be carried on in more or less distinct parts of the cerebral hemispheres. To question this is to ignore the truths of neuro-physiology, as well as those of physiology in general. It is proved experimentally, that every bundle of nerve-fibers and every ganglion has a special duty, and that each part of every such bundle, and every such ganglion, has a duty still more special. Can it be, then, that in the great hemispherical ganglia alone this specialization of duty does not hold? That there are no conspicuous divisions here is true, but it is also true in other cases, where there are undeniable differences of function—instance, the spinal cord, or one of the great nerve-bundles.

"Just as there are aggregated together in a sciatic nerve an immense number of fibers, each of which has a particular office, referring to some one part of the leg, but all of which have for their joint duty the management of the leg as a whole; so, in any one region of the cerebrum, each fiber may be concluded to have some particular office, which, in common with the particular offices of many neighboring fibers, is merged in some general office fulfilled in that region of the cerebrum.

"Any other hypothesis seems to me, on the face of it, untenable. Either there is some arrangement, some organization, in the cerebrum, or there is none. If there is no organization, the cerebrum is a chaotic mass of fibers, incapable of performing any orderly action. If there is some organization, it must consist in that same 'physiological division of labor,' in which all organization consists; and there is no division of labor, physiological or other, but what involves the concentration of special kinds of activity in special places."

It may be objected by some, in behalf of Dr. Brown-Séquard's doctrine, that the remarks of Mr. Spencer are not really aimed against it. But I am unable to see how the views of the former can be reconciled to the general, and with the exception of the cerebral cortex—if it is an exception—the all but universal principles of physiology. The sensory and motor nerves are distinct. Each nerve, as a rule, whether motor or sensory, has its special peripheral area of distribution, and its special central nucleus and region of termination. Each step in the progress of knowledge of the spinal cord and medulla only serves to render more clear the fact, that special tracts of gray matter of the same are charged with special functions. The same general view begins to be something more than a surmise as regards the basal system of ganglia, upon which the peripheral nervous system converges.

Up to this point, localization of function cannot be denied with any show of reason. But above this point it is denied by Dr. Brown-Séquard. It is assumed as highly probable by those who hold to the localization of function in the cerebrel cortex, that the fibers which arise from the cells of the masses which compose the "basal system," as they pass upward in the *corona radiata* of Reil, connect particular areas of the gray matter of the basal system with particular regions of the cortex, in the same manner as in the peripheral nervous system, the fibers of which connect particular parts of the gray matter of the cord or medulla with the same muscles, etc., as a rule. An impression produced at a particular part of the periphery is conveyed in different individuals (save in anomalous cases) essentially to the same part of the gray matter in the cord. It passes up then by preference, in a certain part of the cord, medulla, crus, etc., into or through a distinct part of the basal system, and from thence along a distinct fiber or bundle of fibers to a definite part of the cortex, even in different animals, as a rule. This view seems absolutely necessary to regularity of action of the nervous system.

But Dr. Brown-Séquard thinks, apparently, that the muscles which move the right hand—or say the index finger—if excited from the cortex are not excited from any special part of it, but from *any* part. There are no *centers* in the cortex, as in all other parts of the nervous system. One cell, for example, which subserves this purpose (if any such cells exist) is to be found in every convolution of the brain. This makes it necessary that one or other or all of certain kinds of nervous mechanisms should exist in the brain. These cells must be either all connected together, or they must be distinct *inter se.* According to Dr. Brown-Séquard most probably the latter is true. This admitted, one of two other suppositions must be true. Either the fibers from each of these widely separated cells must make their way independently to the same part of the basal system, or to different parts of the same, most likely the latter, according to our author. So that instead of one part, many parts of the basal system would be simultaneously excited, in order to finally move the muscles of the right index finger. But if only *one* cortical cell is excited, instead of many, which one out of the hundreds of cells probably devoted to this purpose shall habitually act? What an infinite complexity of fibers does not this doctrine of Brown-Séquard's render necessary? But if motor cells for the muscles of the index finger are

"scattered broadcast" all through the cerebral cortex, the same must be true for excitor or sensory impressions. Though they may start from some definite peripheral area of the body, as the retina, and be conveyed to a certain part of the basal system—let us suppose—yet if the doctrine of Dr. Brown-Séquard were true, the visual impressions, if they are sent to the cortex, may go to any or all of its parts, for the cells for the final appreciation of visual impressions are disseminated in all parts of the cortex. It would be necessary for the fibers of the optic tract (nerve) after being traced back to a basal nucleus to which they lead, to give off a vast complex of fibers to the cortex, each one of which, if all the others were destroyed, might serve the purposes of visual perception. But what anatomical proof have we of such a state of things as Dr. Brown-Séquard supposes? None whatever. It is simply made possible by our anatomical ignorance.

I have no hesitation in saying, that the anatomical evidence, so far as it is clear, is in favor of a localization of function in the cortex. I am ready to submit the details whenever a suitable opportunity offers, but cannot do so this evening.

The physiological evidence is mainly composed of the results of the researches of Fritsch and Hitzig and Ferrier and many others, now so generally known. They all tend to show the existence of limited cortical areas which for the same animals have peculiar functions. Thus far the results of experiment have been in many respects unsatisfactory, but out of the whole, the general doctrine of a localization of functions in the cortex has clearly emerged. But the conditions of success are numerous and delicate, and are with difficulty complied with. One of the first steps taken, has been the localization of an area lying about the fissure of Rolando, as the so-called "motor zone." From within the rather ill-defined limits of this area, it has been found possible to provoke contractions of various muscular groups by electrical excitation of the cortex—or of the fibres within or immediately beneath it. Particular spots within this area have been fixed on, from which the contractions of special groups of muscles can be excited *on the opposite side* of the body. These definite spots have been called "psycho-motor" centers. They have been imagined, on pretty good grounds, as containing a group of motor cells worthy of being called a nervous center, and from which the *will* acts, in the exercise of *volition*, to excite a definite *purposive* contraction. There must be some part of the brain from which such actions may be excited. The experiments touching this subject, conducted on the higher animals—especially monkeys—have now become so numerous and critical as to leave one in a state of surprise, when they consider the utterances of Dr. Brown-Séquard and a few others, chiefly his pupils, such as my friend Dr. Dupuy. For some of the details I must refer you more particularly to the work of Dr. Ferrier, republished in this country—a work all should carefully read, who seek information on this subject. Particular spots have been designated by Dr. Ferrier and others, within the "motor zone," excitation of which provoke with singular regularity contractions of special muscular groups on the opposite side of the body. Destruction of the same spots have with almost equal uniformity led to paralysis or pareses of the same muscles. To enumerate and critically discuss this phase of our subject adequately, would require a course of lectures. But in the presence of existing information, I am willing to make the prediction that before five years shall pass, no doubt will exist in the mind of any well-informed and unprejudiced physician as to the truth of the doctrine, against which Dr. Brown-Séquard has raised his voice, in his course of lectures in this city, and in other lectures and writing delivered elsewhere.

I have now to mention that quite recently, Drs. Bevan Lewis, and Henry Clarke of London, have begun to supply the minute anatomical evidence of the correctness of the doctrine of a localization of function in the cerebral cortex. In a paper (January last) in the *Proc. Roy. Soc.*, London (and in another highly interesting paper in "*Brain*," a recent periodical, published from London,) they have described the "giant or motor cells" of the cortex, of Betz, found within the "motor zone." They find them chiefly confined to this zone, but gathered *into constellations or centers*, especially at the exact spots located by Ferrier as the seat of motor centers. Their anatomical discoveries, taken in connection with those of Ferrier, Hitzig and others, are certainly remarkable. I cannot discuss, this evening, either the facts which seem to support, or the difficulties (for there are difficulties) of this doctrine. But I cannot see how, in view of the anatomical and physiological evidence at present in existence on this subject, any one can reasonably assume the position of Dr. Brown-Séquard.

As to the pathological evidence it is becoming abundant and is rapidly on the increase. Cases of limited lesions of sense and motility in man, in which examinations post mortem, have revealed demonstrable material lesion of the cortical centers, presumably governing such functions or parts are now very numerous and many of them critical on account of the application to them of the best methods known and by thoroughly competent hands. The best collection of such cases is in a recent number of the *Rivista Speriment. di Freniatria, etc., anno* iv, *Fascicolo* 1., p. 1, 1878. (*Le Localizzazioni Motrici della Cortecci Cerebrale studiate speciale dal lato Clinico, dal dott. Maragliano*). Ninety-seven cases are collected from various sources and analyzed in this paper, and I must say when they are compared with the heterogeneous and uncritical collection of cases of Dr. Brown-Séquard, of lesion of the brain, either with absence of symptoms, or with different symptoms, that the disparity is enormous, as to scientific value, the preponderance being wholly on the side of the cases which support the doctrine of a localization of function in the cortex.

This subject has been discussed in a multitude of papers from the best hands, especially within the past year or so, and the result of the whole is, so it seems to me, to place the general doctrine on a permanent basis. But there are yet special difficulties, many of which have been industriously used (and I had almost said misused) by Dr. Brown-Séquard in his recent lectures in this city. But I have no doubt they will be removed in due time.

Dr. Brown-Séquard relies on certain experiments on the lower animals, but especially on pathological cases, to support his doctrine of non-localization of function in the cortex. But I have no hesitation in declaring that, as compared with the evidence on the other side, whether physiological or pathological, it is worthless.

Quite recently Dr. Ferrier has discussed this subject afresh in a course of lectures, now being published in the *British Medical Journal.* In these lectures he has undertaken to answer many of the objections of Dr. Brown-Séquard, and in a manner which must be satisfactory to nearly all, except the latter.

I would be glad, if I had time, to discuss Dr. Brown-Séquard's use (abuse as I was about to say) of the principle of *inhibition* and its contrary, in explaining paralysis and convulsions from brain disease. No one disputes intelligently that we may and do have *inhibitory paralysis.* Dr. Brown-Séquard cannot be permitted to monopolize this principle (of inhibition), which he was not the first to notice and apply. No one objects to this principle, or to admitting it as a factor in paralyses, especially from brain disease. But Dr. Brown-Séquard contends in its favor, as if he had a host of opposers, or as if he alone among physiologists admitted it. Among the real objections to his mode of using this principle in explaining paralyses from disease of the brain, is this: His gratuitous use of the principle of inhibition to explain how it comes to pass that disease in one side of the brain produces paralysis in the opposite side of the body, in such way as to preserve his assumption in regard to the non-decussation of the motor conductors. He sacrifices a multitude of apparent facts, at the point of a bare and unprovable assumption. In doing so, Dr. Brown-Séquard has apparently been bereft of all his former candor and caution in reasoning. His "cases" are *not* critical; they do not satisfy the reasonable demands of scientific proof. But I can do little more than challenge the positions taken by the lecturer. It may be that I can hereafter refer to this subject in papers before the society, if opportunity should offer. But it is now being so amply discussed by various competent persons that the time cannot be distant when all reasonable doubts will be removed, and the doctrine of a localization of function in the cortex become a recognized principle in nervous physiology.

